# AUDIT^2 – Identification and Management of Alcohol Use Problems in the Southern Gambling Service: A Full Audit Cycle

**DOI:** 10.1192/bjo.2026.11181

**Published:** 2026-06-30

**Authors:** Yasmin Mohamed Yousof, Konstantinos Ioannidis, Filipa M.A.A. Teixeira, Sam Chamberlain

**Affiliations:** 1 Hampshire and Isle of Wight NHS Trust, Southampton, United Kingdom; 2 South West London and St George’s Mental Health NHS trust, London, United Kingdom

## Abstract

**Aims:**

The re-audit aimed to assess compliance with previously implemented recommendations regarding alcohol assessment, intervention, documentation, and signposting for people with gambling disorder presenting to the Southern Gambling Service (SGS), and to evaluate improvements in practice since the initial audit.

**Methods:**

The re-audit included all referrals received by the Southern Gambling Service (SGS) between 1 March 2025 and 30 September 2025 who completed an initial assessment.

Baseline data were analysed to stratify patients’ alcohol use risk based on their extended Alcohol Use Disorder Identification Test (AUDIT-C) and Estimated Weekly Alcohol Consumption (EWAC), collected via a digital pre-assessment tool, with escalation to a full AUDIT where indicated. Clinical assessment letters and formulations were reviewed to assess documented compliance with National Institute for Health and Care Excellence (CG115) guidance, Department of Health and Social Care guidance, and Royal College of Physicians regarding appropriate management according to their risk brackets. Outcomes included: completion of full AUDIT for those meeting criteria; delivery and documentation of brief interventions for hazardous or harmful drinking; provision of advice on avoiding abrupt reduction for those scoring ≥20; delivery and documentation of formulations on alcohol use and signposting to specialist alcohol services where appropriate.

**Results:**

A total of 301 referrals were received during the audit period, of which 124 referrals (41.2%) were accepted and assessed. AUDIT-C and EWAC were completed for 100% of assessed patients. Fifty-five patients (44.4%) met criteria for escalation and completed a full AUDIT. Of these, 27 patients (49.1%) scored 8 or above, indicating hazardous or harmful drinking, and 10 patients (18.2%) scored 20 or above, suggesting possible alcohol dependence. Among patients scoring ≥8, 18 (66.7%) received a documented brief intervention and 20 (74%) had an alcohol-related formulation recorded. Of those scoring ≥20, seven (70%) received documented advice on avoiding abrupt alcohol reduction and eight (80%) had a formulation addressing alcohol use. Signposting to specialist alcohol services was documented for 7 of 10 (70%) patients who met criteria.

**Conclusion:**

The re-audit demonstrates substantial improvements across all areas compared with the initial audit. Full AUDIT completion improved from 0% to 100%, delivery of brief interventions from 7% → 67%, advice on safe alcohol reduction from (0% → 70%) and signposting for patients improved substantially. Maintaining current standards with improvement in documentation consistency, staff education and reinforcement of intervention thresholds can help improve these gains.

